# Effects of Fluoroquinolones on Aortic Aneurysm or Dissection Processes: A Systematic Review and Meta-Analysis

**DOI:** 10.31083/RCM43656

**Published:** 2026-03-06

**Authors:** Zhi-Yuan Wu, Yang Yang, Zhao-Long Li, Wen-Xin Zhao, Zuo-Guan Chen, Yong-Peng Diao, Yong-Jun Li

**Affiliations:** ^1^Department of Vascular Surgery, Beijing Hospital, National Center of Gerontology; Institute of Geriatric Medicine, Chinese Academy of Medical Sciences, 100010 Beijing, China; ^2^Institute of Molecular Vascular Medicine, Technical University Munich, 80331 Munich, Germany; ^3^Peking University Health Science Center, 100010 Beijing, China; ^4^Peking Union Medical College, Chinese Academy of Medical Science, 100010 Beijing, China

**Keywords:** aortic aneurysm, fluoroquinolones, antibiotics, adverse reactions, meta-analysis

## Abstract

**Background:**

This systematic review/meta-analysis investigated the risks of fluoroquinolones (FQs) for aortic aneurysms (thoracic/abdominal) and Stanford A/B dissections.

**Methods:**

We searched EMBASE, Ovid, PubMed, Web of Science, and Scopus databases in February 2024. Eligible observational studies were those that presented adjusted risk estimates for aortic aneurysm or dissection (AAD) incidence, aortic-specific mortality, or all-cause mortality in FQ-treated versus untreated unexposed populations.

**Results:**

A total of 13 studies were included (36,224,419 participants), eight of which were cohort studies, two were nested case-control studies, and three were case-crossover designs. FQ exposure was associated with significantly elevated *de* *novo* AAD risk within 30 days (relative risk (RR) = 3.40, 95% confidence interval (CI) = [2.72, 4.24]; heterogeneity: *I*^2^ = 41.5%, *p* = 0.11) and 60 days (RR = 3.53, 95% CI = [2.78, 4.49]; heterogeneity: *I*^2^ = 87.0%, *p* < 0.0001). The analysis also revealed a higher all-cause mortality risk for FQs versus non-exposed controls (odds ratio (OR) = 1.44, 95% CI = [1.08, 1.93]; heterogeneity: *I*^2^ = 0%, *p *= 0.80). Subgroup analysis demonstrated comparable aortic dissection (AD) and aortic aneurysm (AA) risks, except for a significantly increased *de* *novo* AA risk at 30 days (RR = 9.13, 95% CI = [6.05, 13.78]; heterogeneity: *I*^2^ = 68.7%, *p* = 0.07) and 60 days (OR = 1.69, 95% CI = [1.27, 2.26]; heterogeneity: *I*^2^ = 52%, *p* = 0.10).

**Conclusion:**

This meta-analysis found a significant association between FQ use and short-term AAD risk. These results suggest that clinicians should weigh the risks of AAD before prescribing FQs, especially in patients with aortic vulnerability or pre-existing aortic pathology, considering alternative treatments when feasible.

**The PROSPERO Registration:**

CRD42024509853 (https://www.crd.york.ac.uk/PROSPERO/view/CRD42024509853).

## 1. Introduction

Fluoroquinolones (FQs) (e.g., ciprofloxacin, levofloxacin, ofloxacin, moxifloxacin) rank among the most prescribed antibiotics worldwide and are used to treat a broad spectrum of infections [1, 2]. Thus, owing to the known broad-spectrum antimicrobial activity and favorable pharmacokinetic properties, FQs have experienced a surge in global prescriptions. Despite widespread use, FQs have been linked to collagen- associated adverse events, including tendinopathy and Achilles tendon rupture [3, 4]. This potential for excessive collagen degradation has raised concerns about arterial wall damage and related adverse events. Aortic aneurysm (AA) and dissection (AAD), among the most lethal cardiovascular conditions, show strong epidemiological associations with male sex, advanced age, hypertension, and a positive family history [5, 6]. Recent studies using large-scale administrative data have demonstrated a significant association between exposure to FQs and an elevated risk of AAD [7, 8, 9, 10, 11, 12, 13, 14, 15]. Given the life-threatening nature of AAD and the increasing utilization of FQ, we conducted this systematic review and meta-analysis to evaluate FQ-associated AAD incidence and clinical outcomes.

## 2. Materials and Methods

### 2.1 Study Design

We had registered the analysis protocol in the International Prospective Register of Systematic Reviews. This work has been reported in accordance with the Preferred Reporting Items for Systematic Reviews and Meta-Analyses (PRISMA) [16] and the Assessing the Methodological Quality of Systematic Reviews (AMSTAR) guidelines [17]. Our primary objective was to evaluate the impact of FQ exposure on the prognosis of *de novo* AAD. Using the PICO (Population, Intervention, Comparison, Outcome) framework, we selected studies that met the following criteria: Population: Cohort participants without pre-existing AAD at baseline; Intervention: systemic FQ antibiotics; Comparison: versus placebo or non-FQ antibiotics; Outcomes: incidence of AAD, aortic rupture, or all-cause mortality [18].

### 2.2 Search Strategy

We systematically searched five databases (EMBASE, Ovid, PubMed, Web of Science, and Scopus) from inception to February 2024. Our search strategy combined two concept clusters: (1) FQs (including besifloxacin, ciprofloxacin, enoxacin, gatifloxacin, levofloxacin, moxifloxacin, norfloxacin, ofloxacin, and pefloxacin); (2) AAD (aortic aneurysm or dissection). The search used the following Boolean structure: (FQs OR [individual FQ agents]) AND (aortic aneurysm OR aortic dissection). Additionally, we manually screened reference lists from included studies and related meta-analyses to identify potentially relevant publications.

### 2.3 Inclusion and Exclusion Criteria

The inclusion criteria for studies included those that (1) enrolled 10 participants per exposure group; (2) performed imaging at baseline (immediately before or concurrent with first exposure to the study drug), with follow-up commencing from this time point; (3) possessed an intervention group that received ≥1 FQ prescription or reimbursement; (4) compared AAD incidence and aortic-specific/all-cause mortality between FQ-treated patients vs. controls (non-FQ antibiotics or no antibiotics). The exclusion criteria comprised: (1) case reports, case series, reviews, editorials, non-original research, or conference abstracts without full data; (2) studies with >75% missing data for predefined variables; (3) duplicate patient populations (only the most recent study retained unless outcomes were complementary).

### 2.4 Literature Screening and Data Extraction

**Fig. 1.**
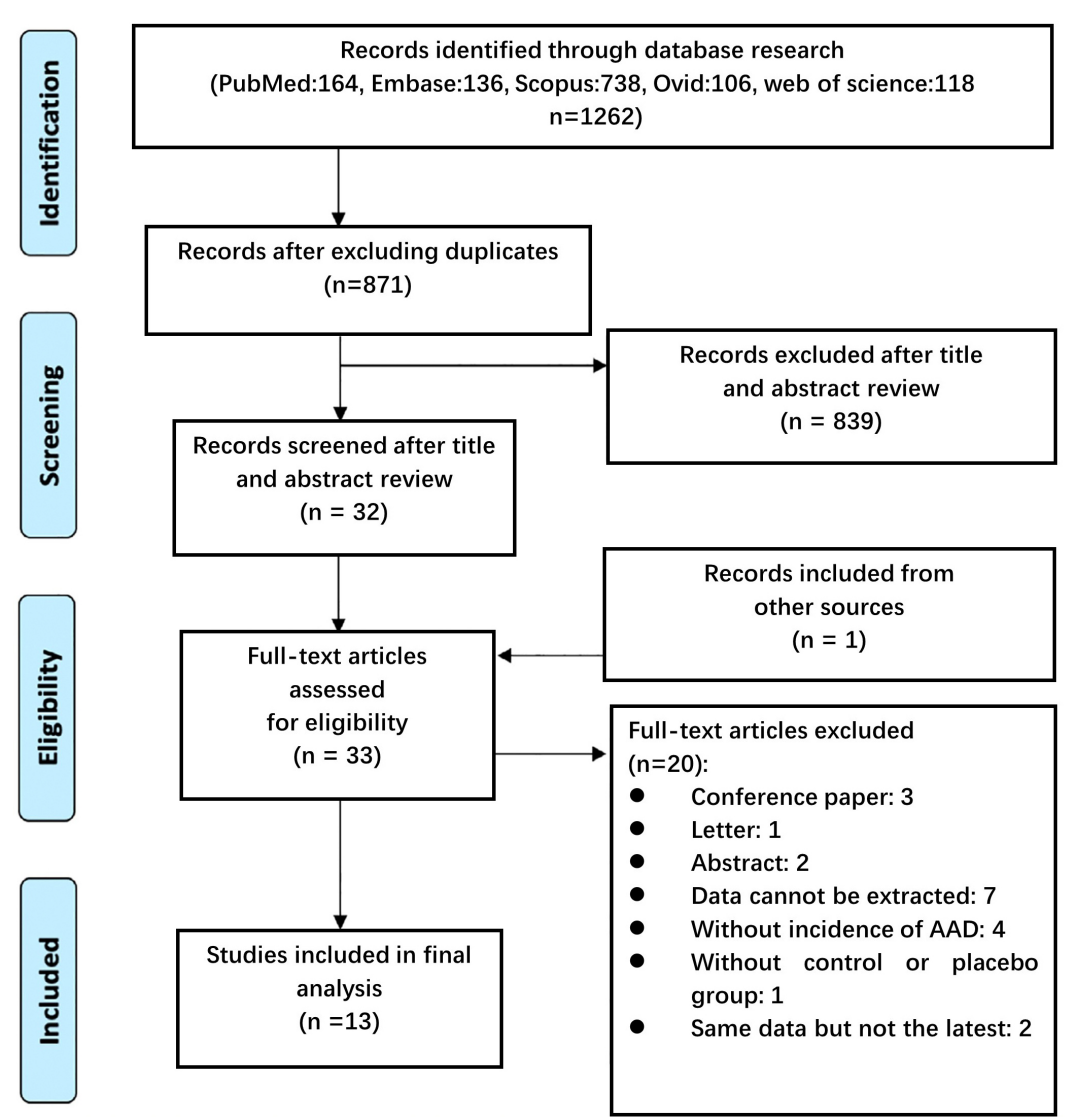
**Flow diagram of the Preferred Reporting Items for Systematic Review and Meta-Analysis guidelines to illustrate the search and selection process during the initial stages of our review**. AAD, aortic aneurysm or dissection.

After removing any duplicate citations, two independent reviewers screened the remaining titles/abstracts. Studies deemed potentially eligible underwent a full-text review, with discrepancies resolved by consensus or third-reviewer adjudication (Fig. 1). A structured extraction form was employed to systematically record study details (e.g., lead author, publication date, and methodology), demographic information, treatment protocols, duration of follow-up, and clinical endpoints (aortic-related deaths, overall mortality, and aneurysm rupture). The co-investigators independently validated all extracted data to ensure accuracy.

### 2.5 Risk of Bias Assessment

Methodological quality was assessed using standardized tools: the Cochrane Risk of Bias tool for randomized controlled trials (RCTs) and the Newcastle–Ottawa scale (NOS) for non-randomized studies, evaluating the selection of exposed/unexposed cohorts, comparability of confounding adjustment, and reliability of outcome assessment.

### 2.6 Data Synthesis and Statistical Analysis

Our analysis incorporated both narrative synthesis and quantitative meta-analytical approaches. For quantitative synthesis, we set a minimum threshold of three comparable studies for meta-analysis. Before pooling data, we evaluated clinical homogeneity across studies by systematically examining key parameters, including: (1) baseline demographic characteristics, (2) intervention protocols, (3) comparator groups, and (4) outcome measures. Statistical heterogeneity was quantified using the *I*^2^ statistic, with interpretation thresholds set as follows: low heterogeneity (*I*^2^ <50%) prompted the use of a fixed-effect model, whereas moderate-to-high heterogeneity (*I*^2^ ≥50%) warranted the use of a random-effects model for the meta-analysis.

The χ^2^ test was used for categorical variables. Random-effects modeling was employed for the pooled analyses to account for potential between-study variability. Between-group differences were considered statistically significant when the 95% confidence intervals (CIs) of the proportions being compared did not overlap. All statistical analyses were conducted in R (version 4.1.0, R Foundation for Statistical Computing, Vienna, Austria) using specialized meta-analysis packages (meta and metafor) to ensure robustness.

## 3. Result

### 3.1 Literature Screening

Of the 1262 initially identified records, 33 articles underwent full-text assessment, and 13 studies met the inclusion criteria regarding evaluating the effects of FQs on AAD outcomes (Table 1 (Ref. [7, 8, 9, 10, 11, 12, 13, 14, 15, 19, 20, 21, 22])). The studies comprised 36,224,419 participants; 8 were cohort studies, 2 were nested case–control studies, and 3 were case–crossover studies. A total of 4 studies were conducted in China [11, 14, 19, 20], 4 in the United States [7, 9, 12, 21], 2 in Korea [8, 22], and the remainder were performed in Sweden [13], Denmark [10], and Canada [15]. Six studies [7, 9, 13, 15, 20, 21] reported associations between the use of FQs and *de novo* AA; five [7, 9, 13, 20, 21] reported associations with aortic dissection (AD);12 [7, 8, 9, 10, 11, 12, 13, 14, 19, 20, 21, 22] reported associations with composite AAD outcomes. A total of 8 studies compared FQs with different antibiotic classes, including 6 with β-lactam antibiotics [9, 11, 12, 13, 19, 22], 4 with macrolides [7, 9, 12, 21], 1 with lincomycin [9], 2 with sulfonamides [9, 12], and 1 with tetracycline [12]. In addition, 4 studies used no-FQs as controls [8, 10, 15, 20]. Table 2 summarizes the key methodological characteristics and exposure profiles of the included studies.

**Table 1.** **Basic characteristics of the included literature**.

First author	Year	Region	Study type	Population	Sample	Intervention	Control	Risk period
Yin‐Yang Chen [19]	2022	Taiwan, China	Cohort study	Patients >18 years and diagnosed with urinary tract infections.	28,568 cases and 28,568 matched controls	Fluoroquinolone	First- or second-generation cephalosporins	90 days
C. C. Lee [14]	2018	Taiwan, China	Case-crossover study	Patients diagnosed with AAD	1213 cases	Fluoroquinolone	Other cardiovascular-related medications	60 days
Yaa-Hui Dong [11]	2020	Taiwan, China	Nested case-control study	Patients ≥20 years	28,948 cases and 289,480 matched controls	Fluoroquinolone	1. Amoxicillin-clavulanate or ampicillin-sulbactam	60 days
							2. Extended-spectrum cephalosporins	
Pei-Han Yu [20]	2020	Taiwan, China	Cohort study	Patients ≤18 years	33,421 cases and 133,684 matched controls	Fluoroquinolone	No-fluoroquinolones	6 months
B. Pasternak [13]	2018	Sweden	Cohort study	Patients ≥50 years	360,088 cases and 360,088 matched controls	Fluoroquinolone	Amoxicillin	60 days
E. R. Newton [9]	2021	USA	Cohort study	Adults aged 18 to 64 years	7,338,704 cases and 24,284,910 controls	Fluoroquinolone	Amoxicillin-clavulanate, azithromycin, cephalexin, clindamycin, and sulfamethoxazole-trimethoprim	90 days
K. Lawaetz Kristensen [10]	2021	Denmark	Case-crossover study.	Patients ≥50 years and diagnosed with ruptured AA	246 cases	Fluoroquinolone	No-fluoroquinolones	28, 60, 90 days
C. Gopalakrishnan [21]	2020	USA	Cohort study	Patients ≥50 years and diagnosed with pneumonia or urinary tract infections	Patients with pneumonia (n = 279,554) or urinary tract infection (n = 948,364)	Fluoroquinolone	Azithromycin	60 days
Nick Daneman [15]	2015	Ontario, Canada	Cohort study	Adults ≥65 years	657,950 cases and 1,086,410 controls	Fluoroquinolone	No-fluoroquinolones	30 days
Sherrie L. Aspinall [12]	2020	USA	Case-crossover study	Veterans ≥18 years who had the outcomes of AAD	127,709 cases	Fluoroquinolone	Azithromycin, doxycycline, cefuroxime, cephalexin, and sulfamethoxazole-trimethoprim	30, 60 days
Nayeong Son [8]	2022	Korea	Nested case–control study	Patients ≥40 years and diagnosed with AAD	29,638 cases and 118,552 controls	Fluoroquinolone	No-fluoroquinolones	60 days
Mahek Garg [7]	2023	USA	Cohort study	Patients ≥18 years	1,587,310 cases and 1,587,310 matched controls	Fluoroquinolone	Macrolides	60 days
Kyungmin Huh [22]	2023	Korea	Cohort study	Patients ≥20 years	158,992 cases and 158,992 matched controls	Fluoroquinolone	Third-generation cephalosporins	1 year

AAD, aortic aneurysm or dissection; AA, aortic aneurysm.

**Table 2.** **Baseline patient characteristics**.

		Number of studies included (n)	Fluoroquinolone group (n, %)		Non-fluoroquinolone group (n, %)		*p*-value
Male		9	7,264,630	(63.46%)	24,637,725	(88.49%)	<0.001
Comorbidities							
	Hypertension	7	3,463,134	(33.07%)	10,069,843	(37.48%)	<0.001
	Coronary heart disease	5	92,077	(0.92%)	175,133	(0.65%)	<0.001
	Cerebrovascular disease	6	111,914	(3.32%)	77,540	(2.70%)	<0.001
	Diabetes	7	1,299,758	(12.41%)	3,436,405	(12.79%)	<0.001
	Chronic obstructive pulmonary disease	6	367,087	(10.02%)	243,165	(7.93%)	<0.001
	Non–large-vessel aneurysmal disease	6	109,397	(1.08%)	61,614	(0.23%)	<0.001
Medication use							
	Antiplatelet	6	303,386	(3.31%)	544,290	(2.14%)	<0.001
	β-blocker	7	1,014,572	(9.43%)	2,378,411	(8.79%)	<0.001

Percentages were calculated based on the number of patients within the FQ group in this study.

### 3.2 Literature Quality Assessment

Methodological quality assessment was performed using the NOS and demonstrated consistently high scores across all included non-randomized studies (Table 3, Ref. [7, 8, 9, 10, 11, 12, 13, 14, 15, 19, 20, 21, 22]). All articles received high scores in the Selection and Compatibility sections, except for Aspinall *et al*. [12], which scored only 2 points due to the potential bias introduced by the study population consisting of veterans. Additionally, most articles did not adequately report follow-up results; only Aspinall *et al*. [12], Lawaetz Kristensen *et al*. [10], and Lee *et al*. [14], avoided this bias owing to the experimental designs employed.

**Table 3.** **Risk of bias**.

Study	Selection				Comparability	Outcome			Total
	Representativeness of the exposed cohort	Selection of the non-exposed cohort	Ascertainment of exposure	Demonstration that the outcome of interest was not present at the start of the study	Comparability of cohorts on the basis of the design or analysis controlled for confounders	Assessment of outcome	Was the follow-up long enough for outcomes to occur	Adequacy of follow-up of cohorts	
Yin‐Yang Chen 2022 [19]	1	1	1	1	2	1	1	0	8
C. C. Lee 2018 [14]	0	1	1	1	2	1	1	1	8
Yaa-Hui Dong 2020 [11]	1	1	1	1	2	1	1	0	8
Pei-Han Yu 2020 [20]	1	1	1	1	2	1	1	0	8
B. Pasternak 2018 [13]	1	1	1	1	2	1	1	0	8
E. R. Newton 2021 [9]	1	1	1	1	2	0	1	0	7
K. Lawaetz Kristensen 2021 [10]	1	1	1	1	2	1	1	1	9
C. Gopalakrishnan 2020 [21]	1	1	1	1	2	1	1	0	8
Nick Daneman 2015 [15]	1	1	1	1	2	1	0	0	7
Sherrie L. Aspinall 2020 [12]	0	0	1	1	2	1	1	1	7
Nayeong Son 2022 [8]	1	1	1	1	2	1	1	0	8
Mahek Garg 2023 [7]	1	1	1	1	2	1	1	0	8
Kyungmin Huh 2023 [22]	1	1	1	1	1	1	1	0	7

Methodological quality assessment with the latest version of the Newcastle-Ottawa scale.

### 3.3 Meta-Analysis Results

**Fig. 2.**
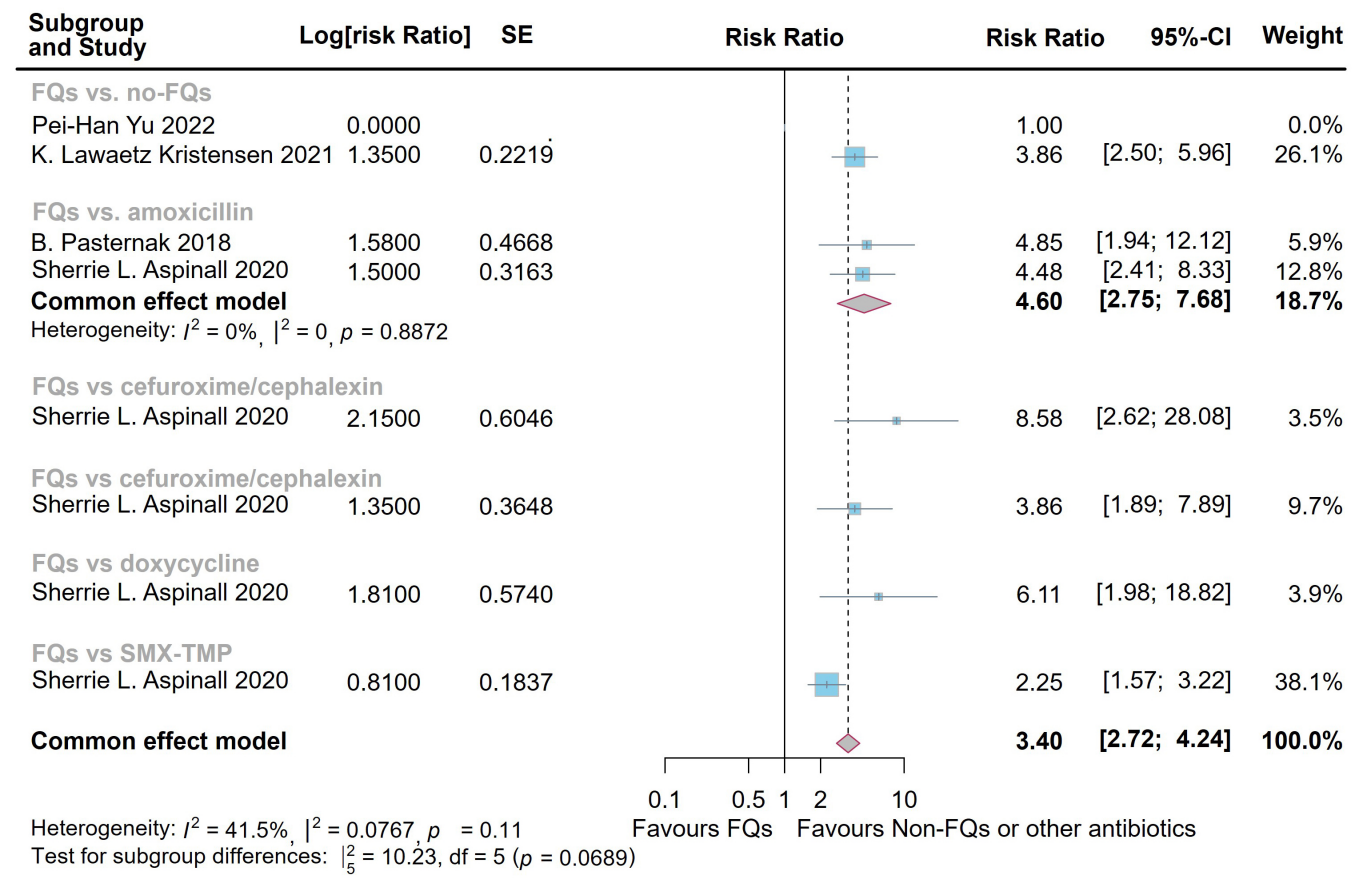
**Forest plot of the risk of aortic aneurysm or dissection (AAD) in the comparison of fluoroquinolones (FQs) vs. controls within a 30-day risk period**. FQs, fluoroquinolones; SMX-TMP, trimethoprim-sulfamethoxazole; SE, standard error; CI, confidence interval.

**Fig. 3.**
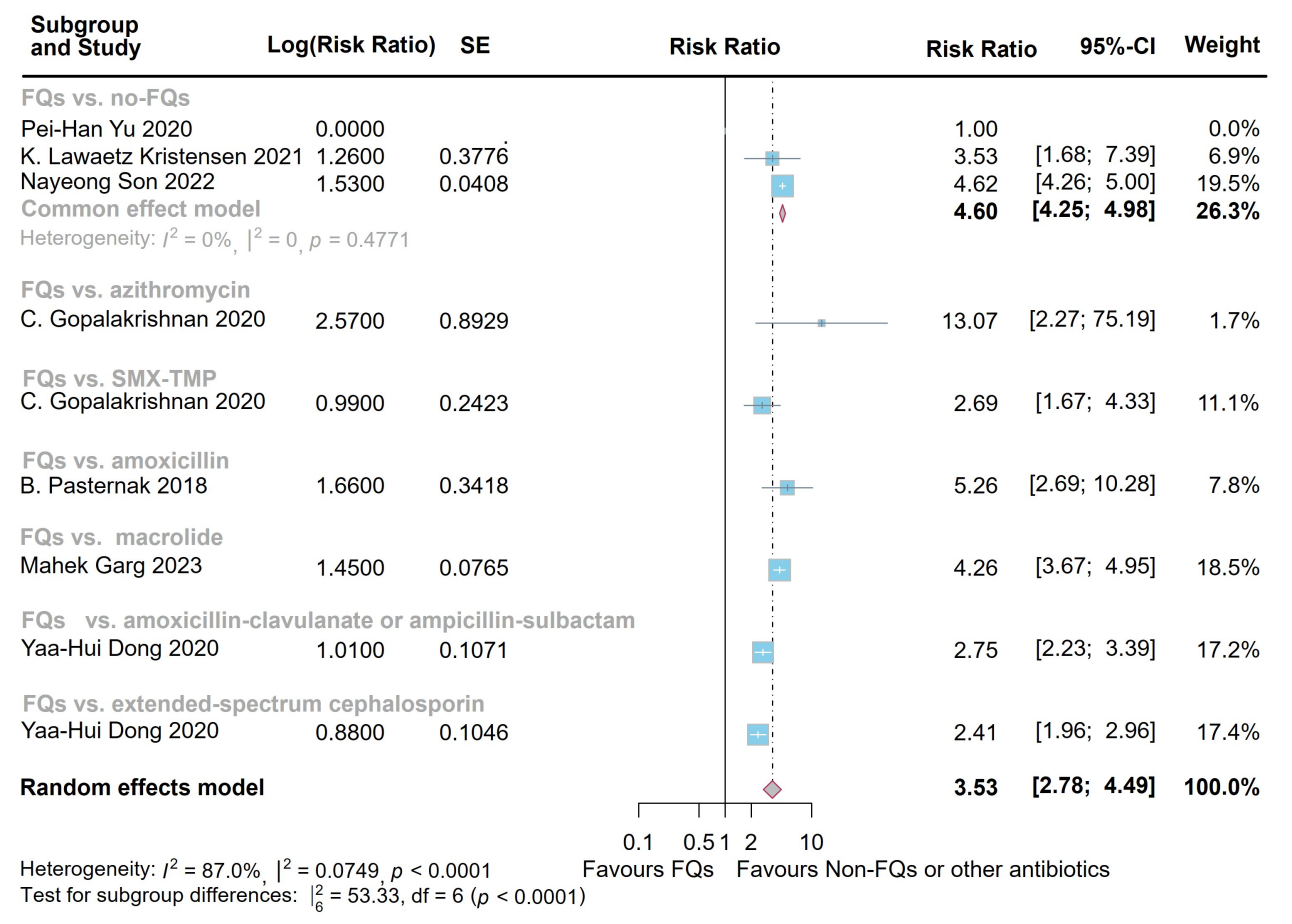
**Forest plot of the risk of aortic aneurysm or dissection (AAD) in the comparison of fluoroquinolones (FQs) vs. controls within a 60-day risk period**. FQs, fluoroquinolones; SMX-TMP, trimethoprim-sulfamethoxazole; SE, standard error; CI, confidence interval.

**Fig. 4.**
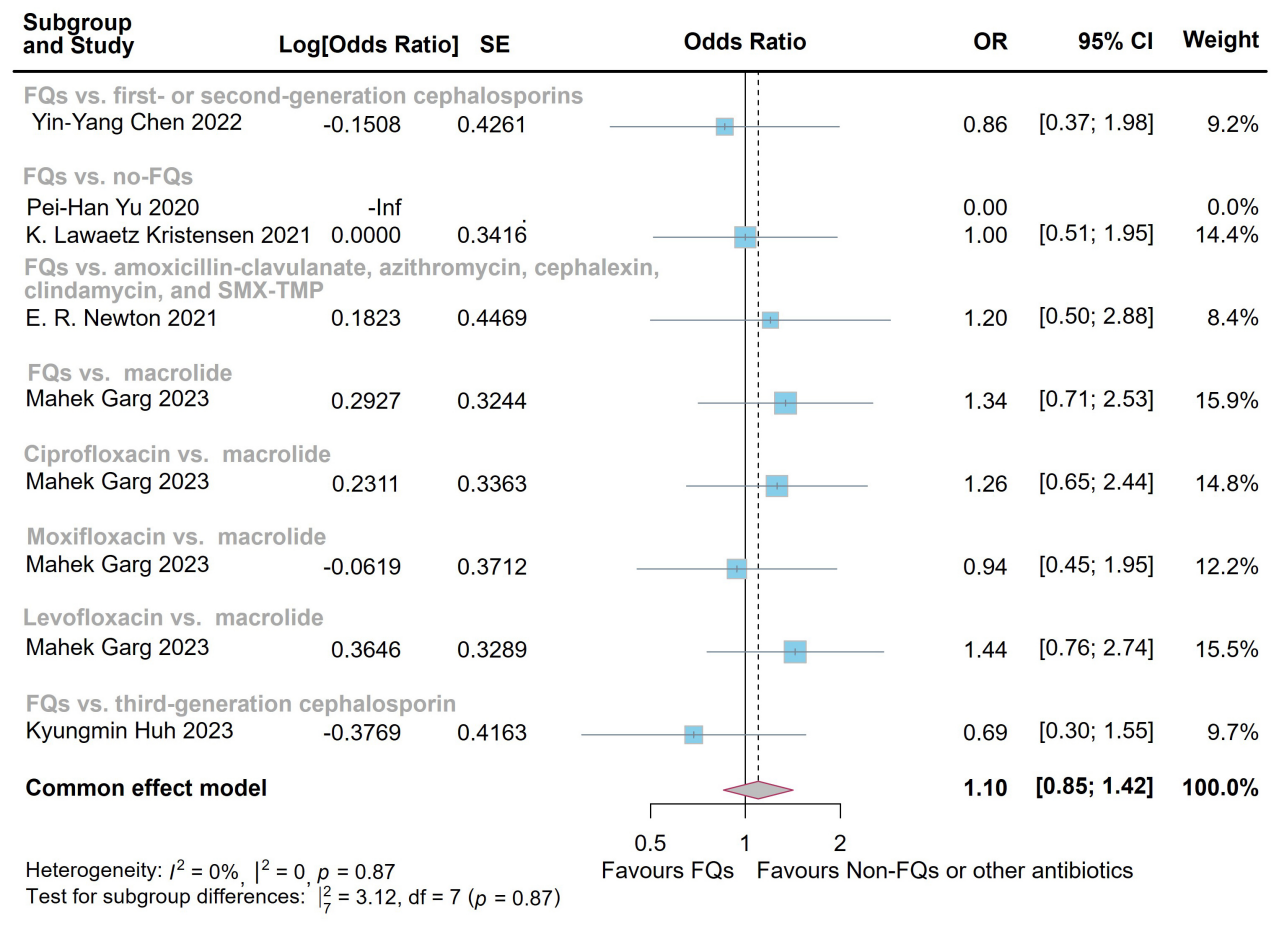
**Forest plot of the risk of aortic aneurysm or dissection (AAD) in the comparison of fluoroquinolones (FQs) vs. controls within a 1-year risk period**. FQs, fluoroquinolones; SMX-TMP, trimethoprim-sulfamethoxazole; SE, standard error; CI, confidence interval.

**Fig. 5.**
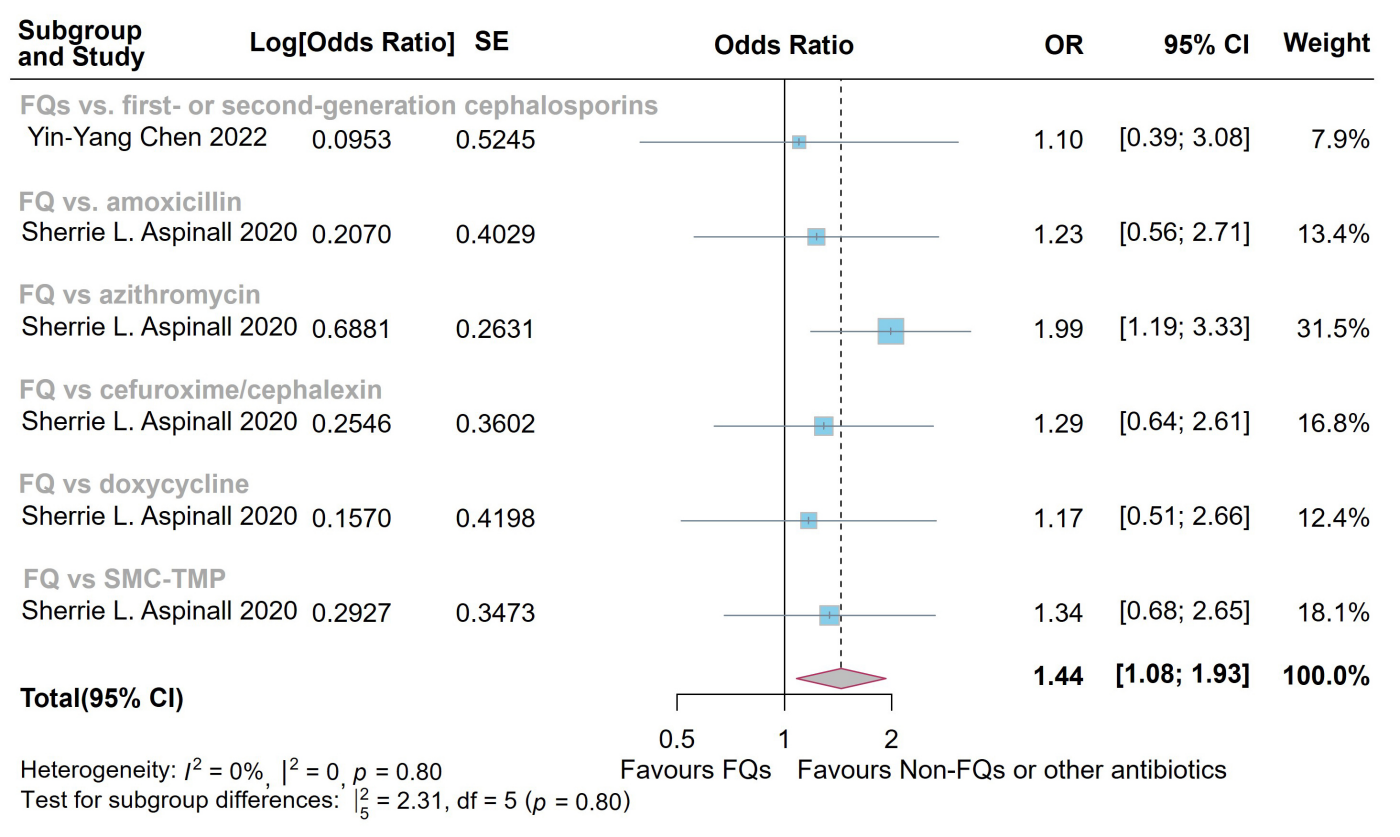
**Forest plot of the risk of all-cause mortality in the comparison of fluoroquinolones (FQs) vs. controls**. FQs, fluoroquinolones; SMX-TMP, trimethoprim-sulfamethoxazole; SE, standard error; CI, confidence interval.

The meta-analysis demonstrated significantly elevated *de novo* risk following the administration of FQs: at 30 days (relative risk (RR) = 3.40, 95% CI = [2.72, 4.24]; heterogeneity: *I*^2^ = 41.5%, *p* = 0.11) and 60 days (RR = 3.53, 95% CI = [2.78, 4.49]; heterogeneity: *I*^2^ = 87.0%, *p* < 0.0001) (Figs. 2,3). However, the difference at 1 year following FQs exposure did not reach statistical significance when compared with controls (OR = 1.10, 95% CI = [0.85, 1.42]; heterogeneity: *I*^2^ = 0%, *p* = 0.87) following FQs exposure when compared with controls (Fig. 4). Two studies [12, 19] evaluated the mortality risk associated with FQs in patients with AAD. The findings of Aspinall *et al*. [12] reported significantly higher mortality risk scores with FQs than with all other antibiotics assessed. This aligns with our meta-analysis that demonstrates increased all-cause mortality risk following FQ exposure (OR = 1.44, 95% CI = [1.08, 1.93]; heterogeneity: *I*^2^ = 0%, *p* = 0.80) (Fig. 5). Meanwhile, subgroup analysis revealed no association between FQ use and the incidence of AD within 1 year of medication (**Supplementary Figs. 1,2**). Additionally, an increased incidence of AA was observed at the follow-up after 30 days (RR = 9.13, 95% CI = [6.05, 13.78]; heterogeneity: *I*^2^ = 68.7%, *p* = 0.07) and 60 days (OR = 1.69, 95% CI = [1.27, 2.26]; heterogeneity: *I*^2^ = 52%, *p* = 0.10) following the use of FQs (**Supplementary Figs. 3,4**); however, no significant correlation was found at the 1-year follow-up (**Supplementary Fig. 5**).

### 3.4 Publication Bias

**Fig. 6.**
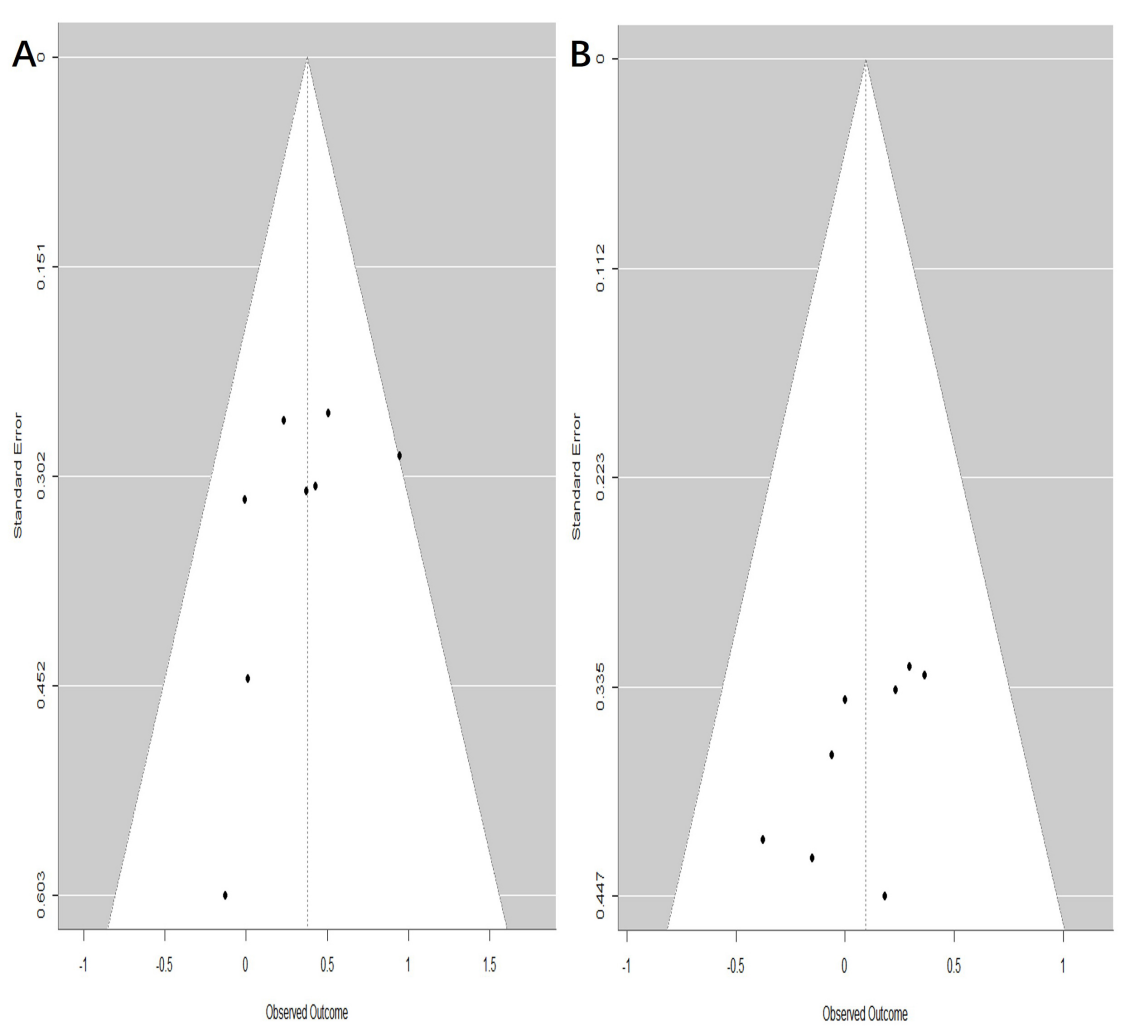
**Funnel plot assessing publication bias of fluoroquinolones (FQs) use with the *de novo* aortic aneurysm or dissection (AAD) incidence at different risk periods**. (A) 60-day risk period; (B) 1-year risk period.

Publication bias was assessed for the outcomes at 60 days and 1 year, with 8 studies included for each time point (Fig. 6). The funnel plots indicated that all points lay within the confidence limits and displayed approximate symmetry, suggesting an absence of significant publication bias.

## 4. Discussion

For many years, clinicians have been seeking interventions to slow the progression of AAD and have evaluated several drugs, including metformin, statins, and antiplatelet drugs. Most drugs did not demonstrate clinically significant efficacy, except for statins and metformin [23, 24]. Meanwhile, the use of antibiotics to slow the progression of AAD was based on the infectious agents identified in plaques; however, most relevant studies have not yielded consistent results [7, 25, 26]. The association of FQs with collagen-disrupting complications (e.g., tendon rupture, retinal detachment) underpins ongoing safety debates regarding the use of FQs in aortic pathologies [27, 28]. Triggered by reported correlations between FQs and aortic pathology [7, 8, 9, 10, 11, 12, 13, 14, 15], the current European Society for Vascular Surgery (ESVS) recommendations have adopted a cautionary stance: FQs remain permissible for small abdominal AAs, but heightened clinical vigilance is required [29].

This meta-analysis synthesizes current evidence demonstrating a significantly elevated AAD risk associated with FQ administration. We demonstrate a time-dependent risk pattern: FQ administration significantly increased short-term (30-/60-day) *de novo* AAD incidence, whereas no statistically significant elevation persisted beyond the initial 60-day period through 1-year follow-up. These findings are consistent with previous studies [10, 13, 22]. A Danish case–-crossover analysis identified a significantly elevated risk of ruptured AA within 28 days of FQ exposure, highlighting a critical vulnerability during antibiotic courses [10]. In addition, Pasternak *et al*. [13] specifically identified a 60-day risk window for incident AA following FQ prescriptions. Conversely, a nationwide cohort study using the Korean National Health Database found similar 1-year AAD risks among patients prescribed oral FQs and third-generation cephalosporins [22]. These findings may relate to the serum half-lives of FQs; thus, we observe a potential bimodal distribution of aortic disease risk after exposure, with increased risk between 30 and 50 days [13, 14, 30]. A similar pattern has been reported for FQ-induced tendon rupture, with onset occurring between 2 and 31 days (median 7 days) [31]. As the half-lives of FQs tend to increase with successive generations, more rigorous studies are needed to clarify the effects of different FQ generations on AAD. Compared with other antibiotics, FQs are associated with an elevated short-term risk of AAD, likely related to the associated acute biological effects and the sudden onset of cardiovascular events. However, the lack of a significant long-term risk difference may be explained by the reversible nature of drug effects, limitations in study methodology, and underlying differences in baseline population risk. Specifically, although the absolute risk remains low, FQs should be prescribed cautiously in high-risk patients (e.g., those with known aortic disease, hypertension, or genetic disorders), and alternative antibiotics should be considered when possible.

The precise pathophysiology underlying FQ-associated aortic complications remains incompletely characterized. However, several biologically plausible mechanisms have been proposed, primarily centered on the disruption of extracellular matrix (ECM) homeostasis in aortic tissue [32]. Indeed, FQs can disrupt the ECM by promoting matrix metalloproteinase (MMP) activation and inhibiting tissue inhibitor of metalloproteinase (TIMP) expression, thereby promoting ECM degradation [32]. FQs also suppress collagen maturation by chelating iron, a cofactor for prolyl 4-hydroxylase and lysyl hydroxylase, essential for collagen cross-linking and strength [33]. Additionally, FQs can induce cell apoptosis and inhibit proliferation in various cell types, which may contribute to aortic destruction [34, 35, 36]. Meanwhile, ciprofloxacin, a common FQ, has been shown to increase MMP expression, decrease lysyl oxidase (LOX) expression, and activate the stimulator of interferon genes (STING) pathway, contributing to aortic wall degeneration [37]. In summary, FQs may increase the risk of AAD by impairing ECM integrity, disrupting collagen synthesis, and inducing cell death.

Interestingly, subgroup analyses revealed comparable effects of FQs on AAD risks, except for *de novo* AA risk at the 30- and 60-day follow-ups. This divergence likely reflects distinct pathogenic pathways: aortic aneurysms primarily result from atherosclerotic degeneration, whereas dissections predominantly stem from collagen defects or inflammatory dysregulation. FQs have also been reported to alter circulating cytokine levels in patients with abdominal aortic aneurysms [38, 39]. When circulating interleukin 6 (IL-6) levels were increased by a 4-week IL-6 infusion in healthy wild-type mice, both macrophage accumulation and abdominal dilation were observed [40]. Meanwhile, collagen degradation depends critically on the enzymatic equilibrium between the lytic activity of the MMPs and the tissue inhibitors of the MMPs. Experimental studies by LeMaire *et al*. [37, 41] revealed that ciprofloxacin promotes aortic dissection pathogenesis in animal models through dual pathways: MMP-9 activation and decreased LOX signaling. However, few studies have examined *de novo* AA and AD outcomes separately, preventing a definitive assessment of differential risk profiles between these pathologies following FQ exposure.

This meta-analysis has several inherent limitations. First, the absence of RCTs restricted the evidence synthesis to observational studies. Second, although most included studies used robust matching methods to reduce confounding, unavoidable systematic biases—particularly exposure misclassification and outcome ascertainment errors—remain due to the fundamental constraints of observational designs. Third, although research on this topic has been conducted worldwide, a significant proportion of reports originates from China and the United States, which may increase the potential impact of regional and racial biases. Furthermore, the small number of studies included in the analysis may not have been sufficient to exclude potential publication bias. Lastly, the clinically oriented outcome categorization (combined AA/AD) used across studies overlooks fundamental pathophysiological distinctions between these conditions—an oversight highlighted by the differential risk patterns observed in our subgroup analyses.

## 5. Conclusion

This meta-analysis identified a significant association between FQs use and short-term risk of AAD. These results suggest that clinicians should carefully weigh the risks before prescribing FQs, especially in patients with aortic vulnerability or pre-existing aortic pathology, and consider alternative treatments when feasible. Further prospective studies are warranted to clarify the mechanisms underlying this association.

## Availability of Data and Materials

Data extracted from included studies, data used for all analyses, analytic code, and other materials used in the systematic review are available upon request from the corresponding author.

## Supplementary Material

Supplementary material associated with this article can be found, in the online version, at https://doi.org/10.31083/RCM43656.
