# Supplementary material for: Effects of Fluoroquinolones on Aortic Aneurysm or Dissection Processes: A Systematic Review and Meta-Analysis
Source: Rev Cardiovasc Med. 2026 Mar 6;27(3):43656. doi: 10.31083/RCM43656 (PMC13036523; doi:10.31083/RCM43656)
Supplement: Supplementary file 1 [file 2153-8174-27-3-43656-s1.zip › Supplementary Figs.docx]

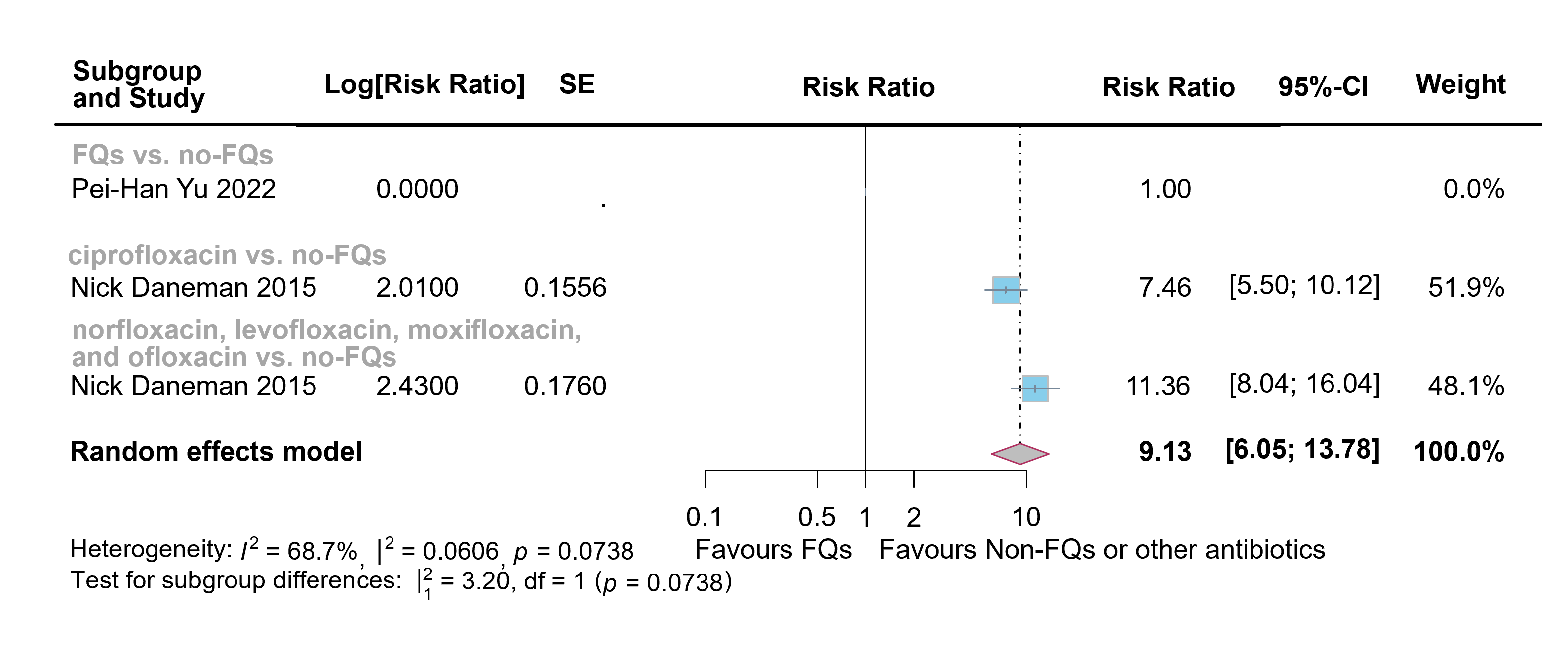


Supplementary Fig. 1. Forest plot of the risk of aortic aneurysm (AA) in the comparison of fluoroquinolones (FQs) vs. controls within a 30-days risk period. FQs, fluoroquinolones; SE, Standard Error; CI, confidence interval.


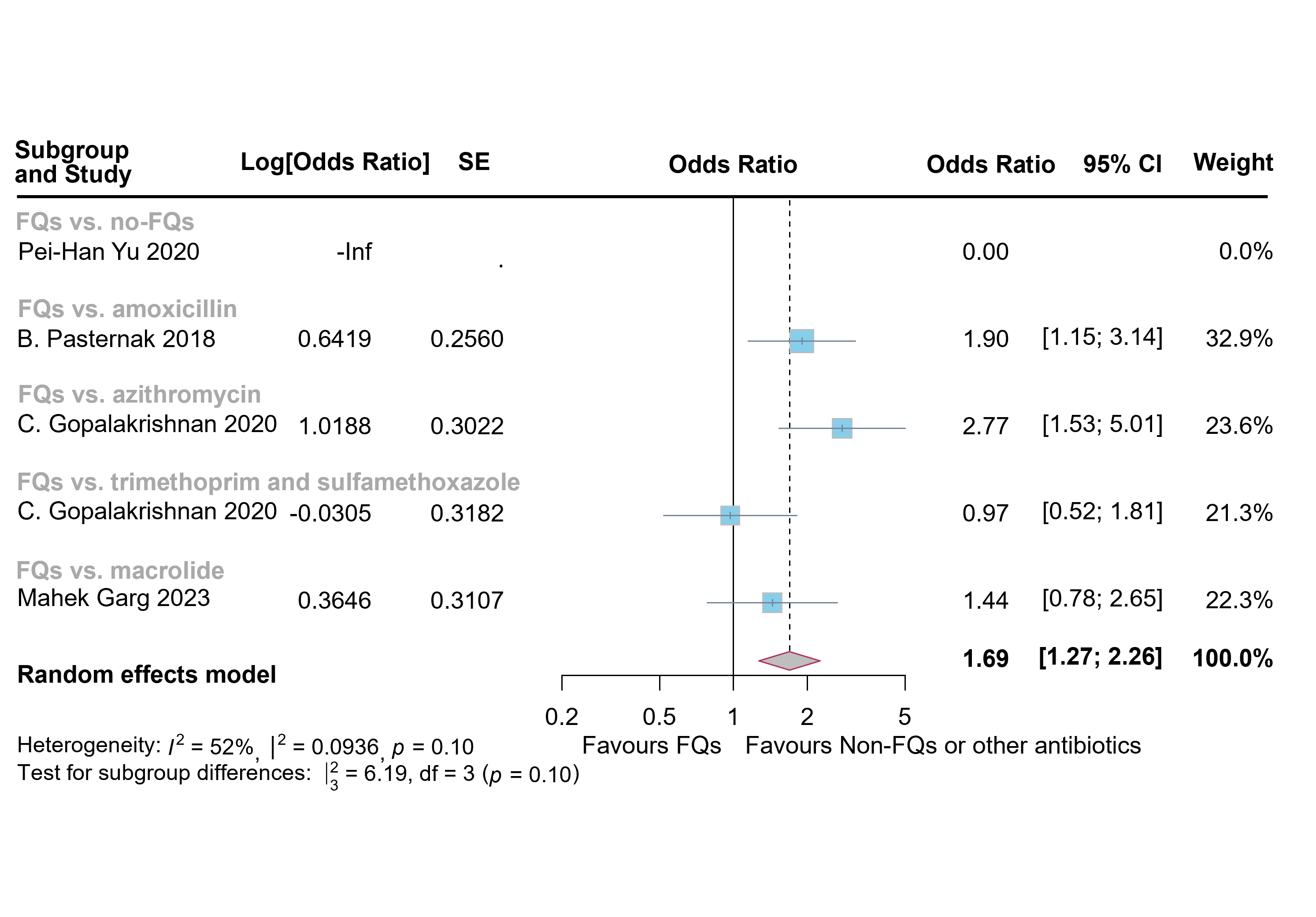


Supplementary Fig. 2. Forest plot of the risk of aortic aneurysm (AA) in comparison of fluoroquinolones (FQs) vs. controls within a 60-days risk period. FQs, fluoroquinolones; SE, Standard Error; CI, confidence interval.


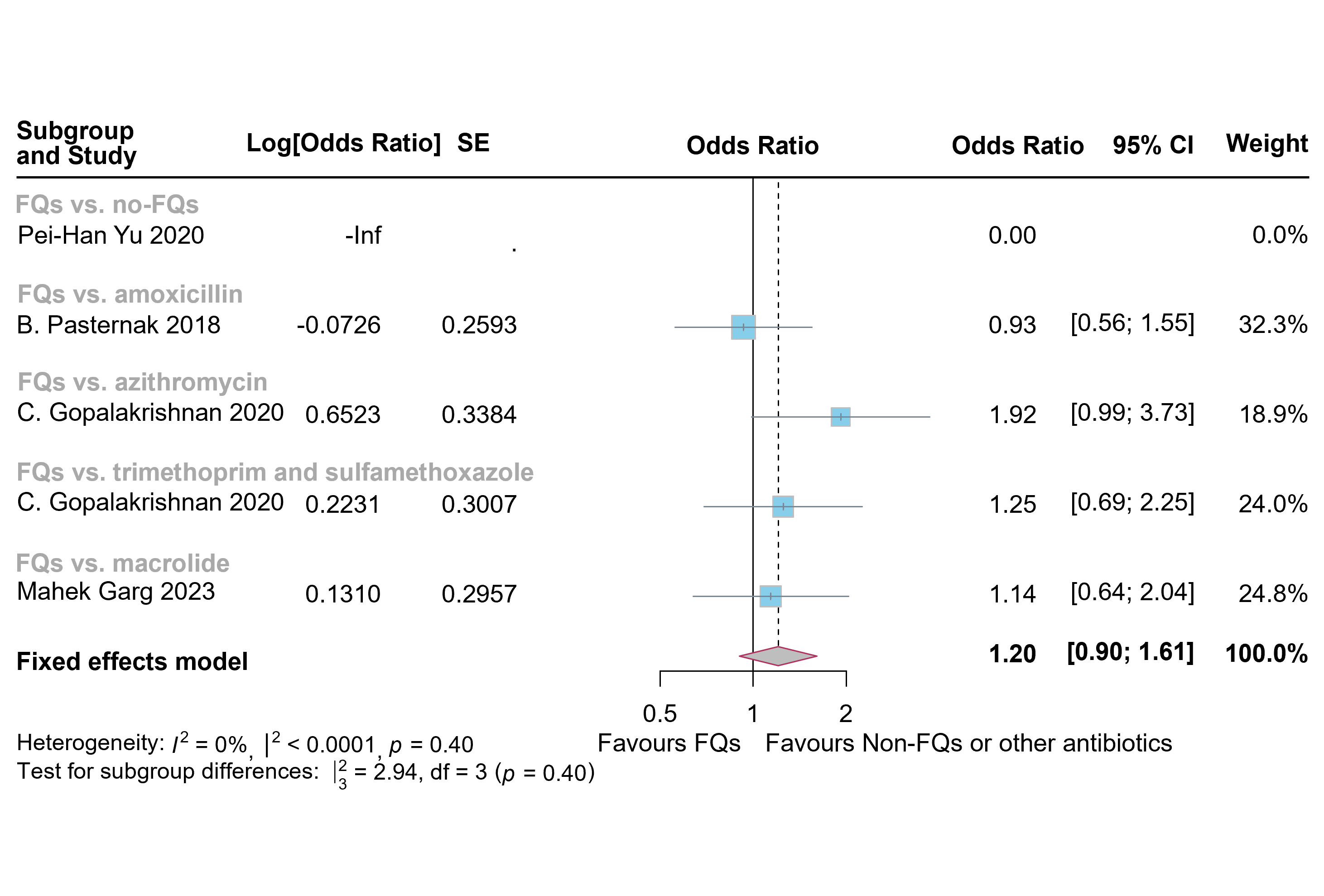


Supplementary Fig. 3. Forest plot of the risk of aortic dissection (AD) in the comparison of fluoroquinolones (FQs) vs. controls within a 60-days risk period. FQs, fluoroquinolones; SE, Standard Error; CI, confidence interval.


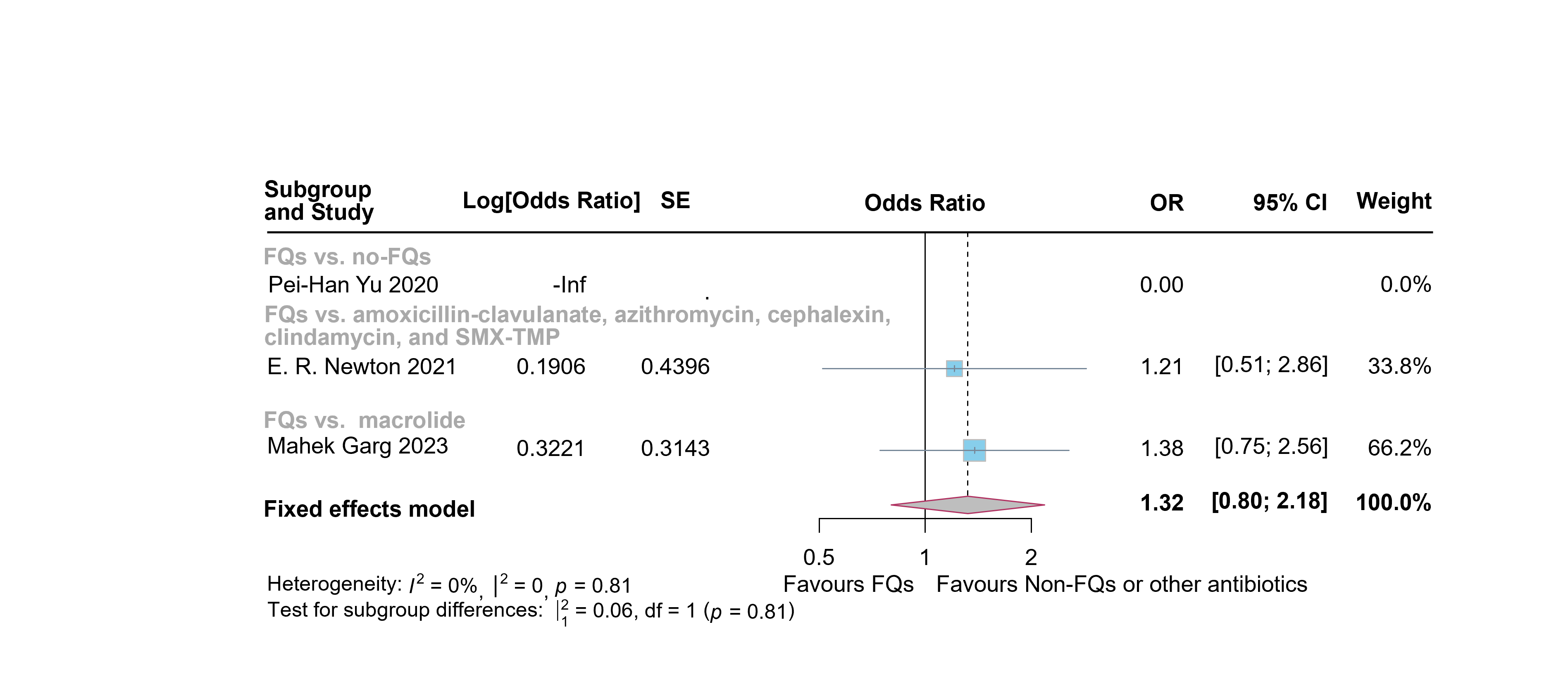


Supplementary Fig. 4. Forest plot of the risk of aortic aneurysm (AA) in the comparison of fluoroquinolones (FQs) vs. controls within a 1-year risk period. FQs, fluoroquinolones; SMX-TMP, trimethoprim-sulfamethoxazole; SE, Standard Error; CI, confidence interval.


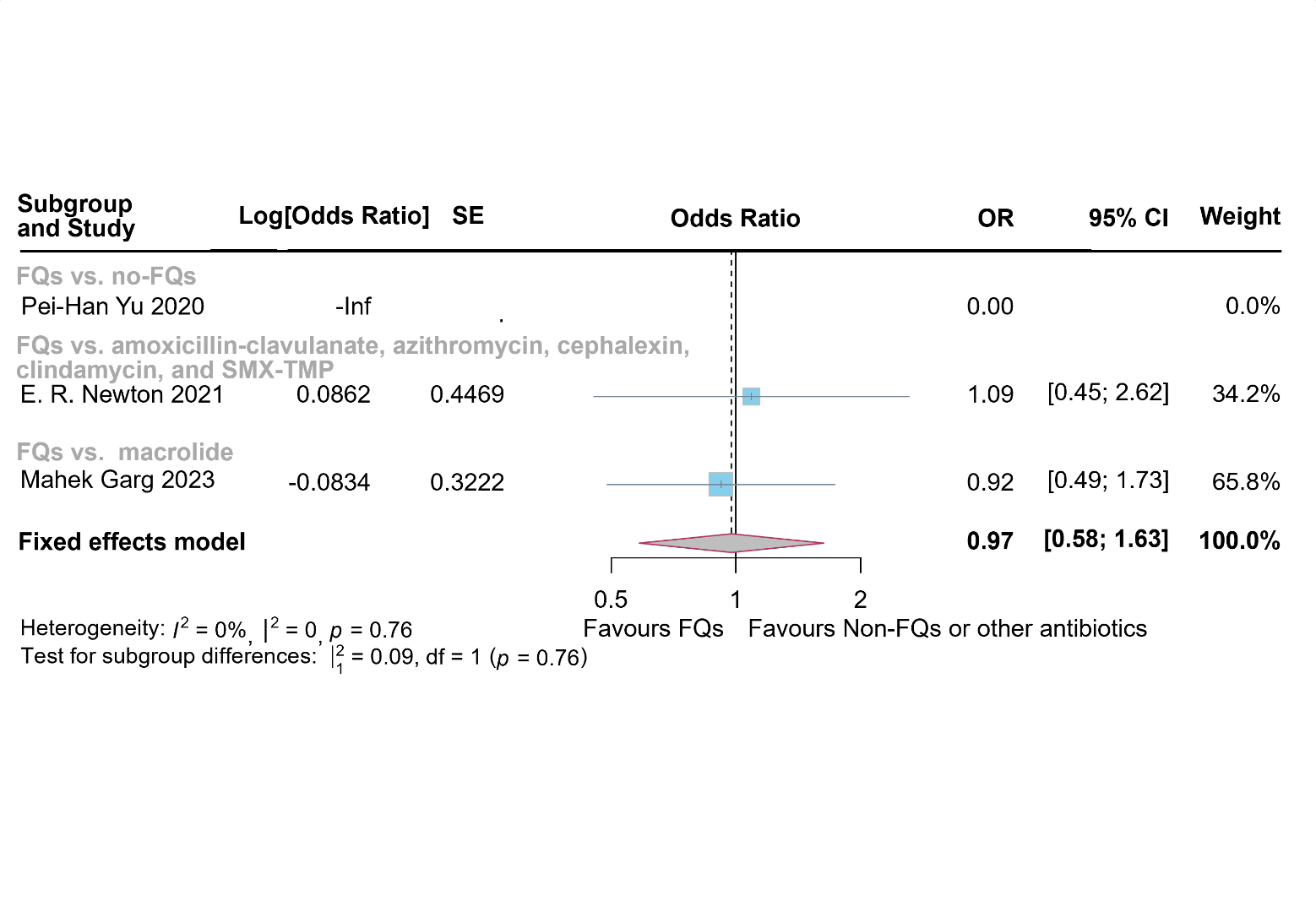


Supplementary Fig. 5. Forest plot of the risk of aortic dissection (AD) in the comparison of fluoroquinolones (FQs) vs. controls within a 1-year risk period. FQs, fluoroquinolones; SMX-TMP, trimethoprim-sulfamethoxazole; SE, Standard Error; CI, confidence interval.
