# Supplementary figures and images for: Effects of Fluoroquinolones on Aortic Aneurysm or Dissection Processes: A Systematic Review and Meta-Analysis
Source: Rev Cardiovasc Med. 2026 Mar 6;27(3):43656. doi: 10.31083/RCM43656 (PMC13036523; doi:10.31083/RCM43656)

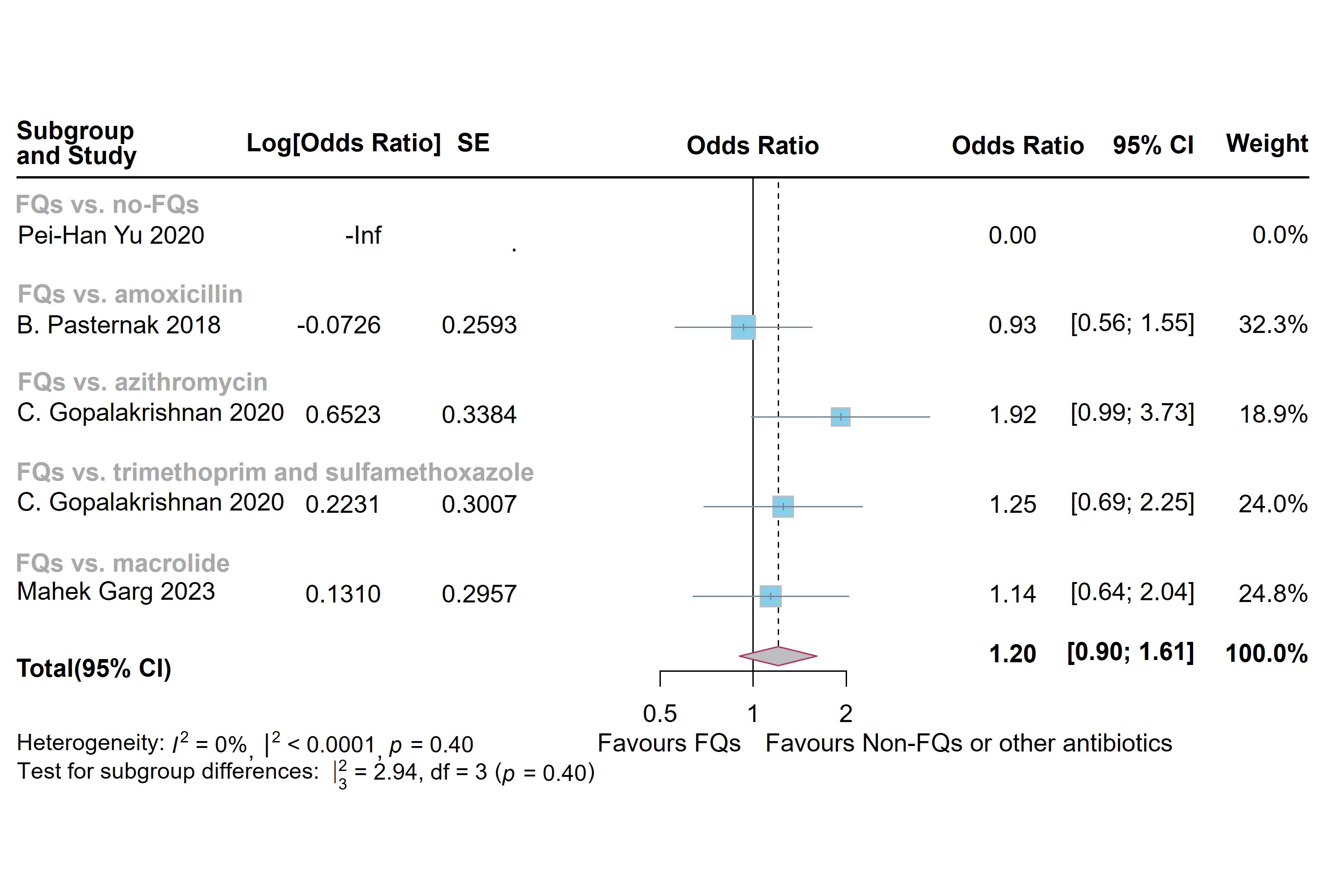

Supplement: Supplementary file 1 [file 2153-8174-27-3-43656-s1.zip › Supplementary Fig. 1.jpg]

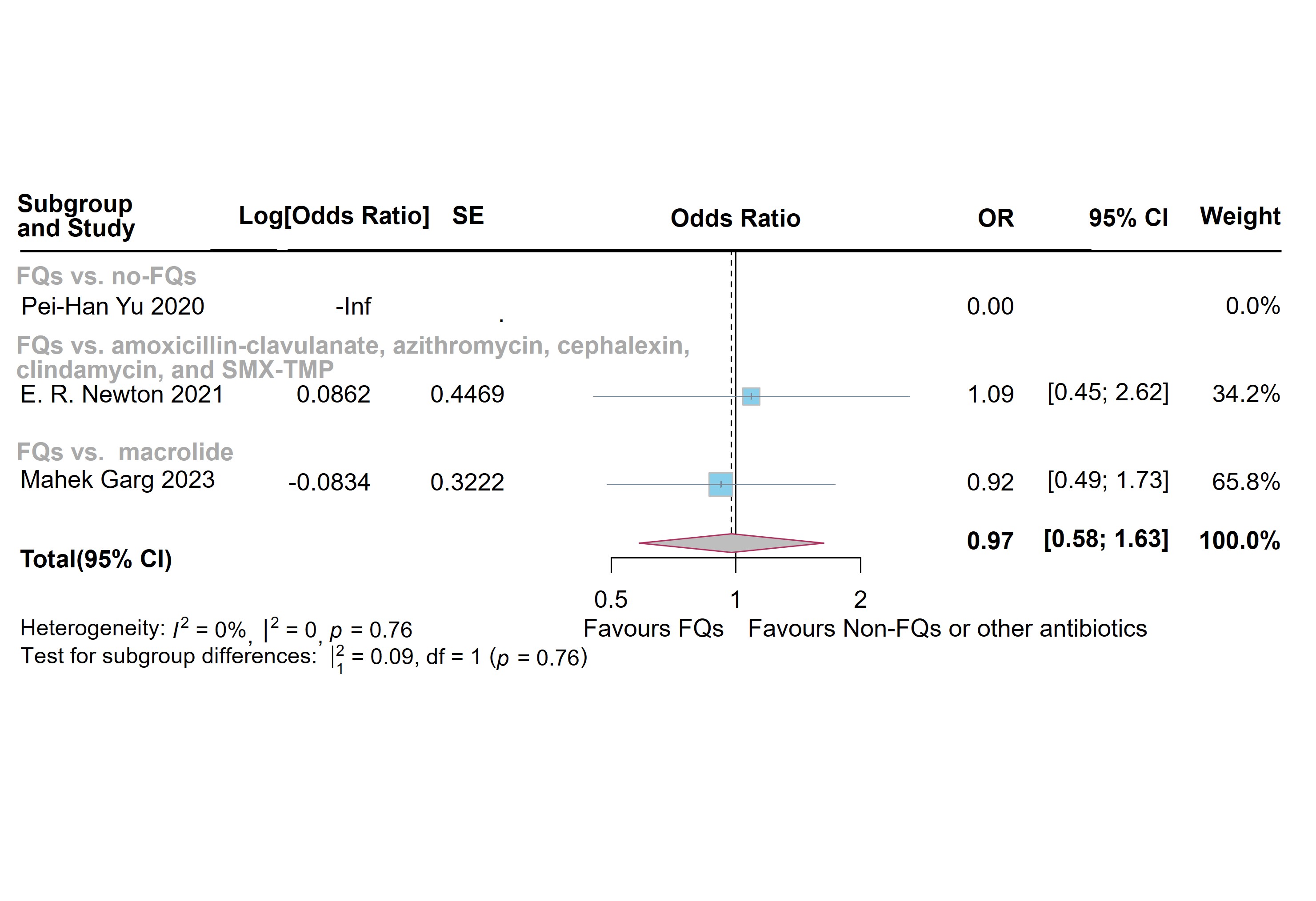

Supplement: Supplementary file 1 [file 2153-8174-27-3-43656-s1.zip › Supplementary Fig. 2.jpg]

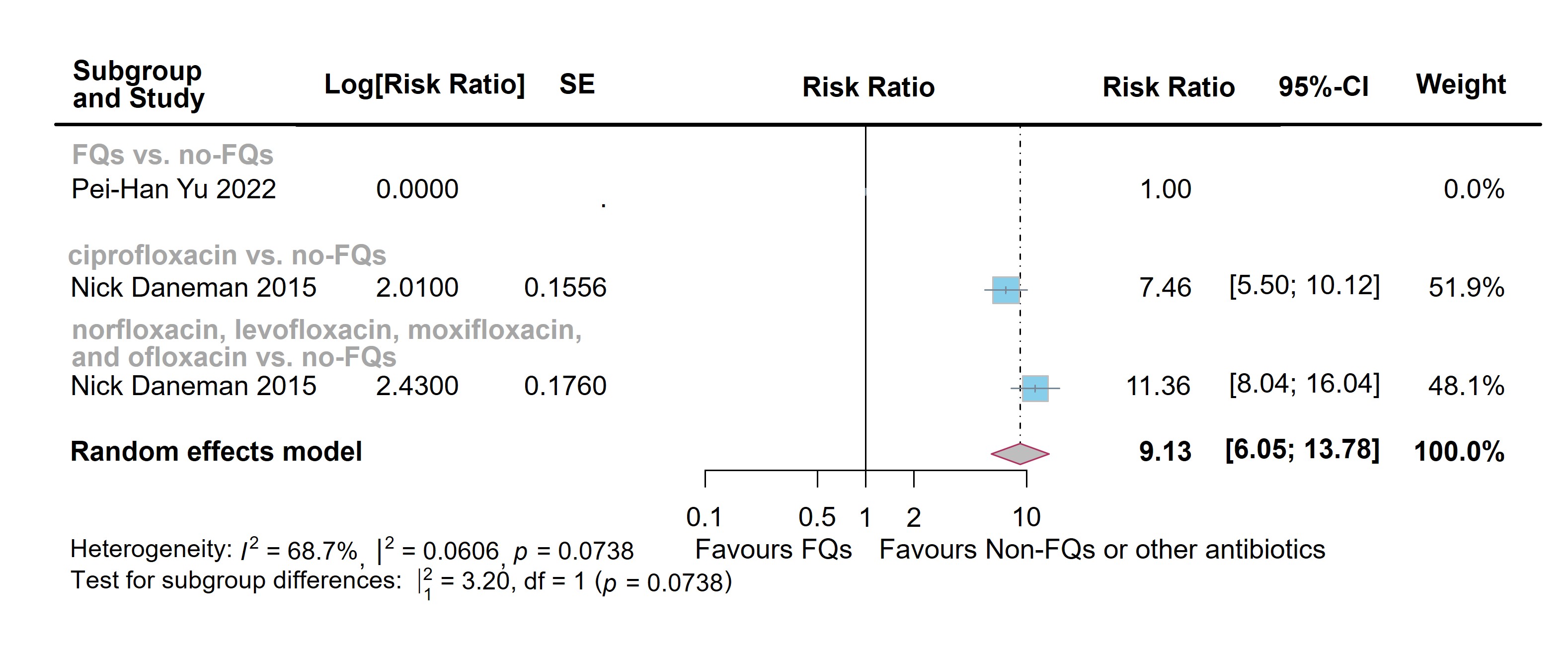

Supplement: Supplementary file 1 [file 2153-8174-27-3-43656-s1.zip › Supplementary Fig. 3.jpg]

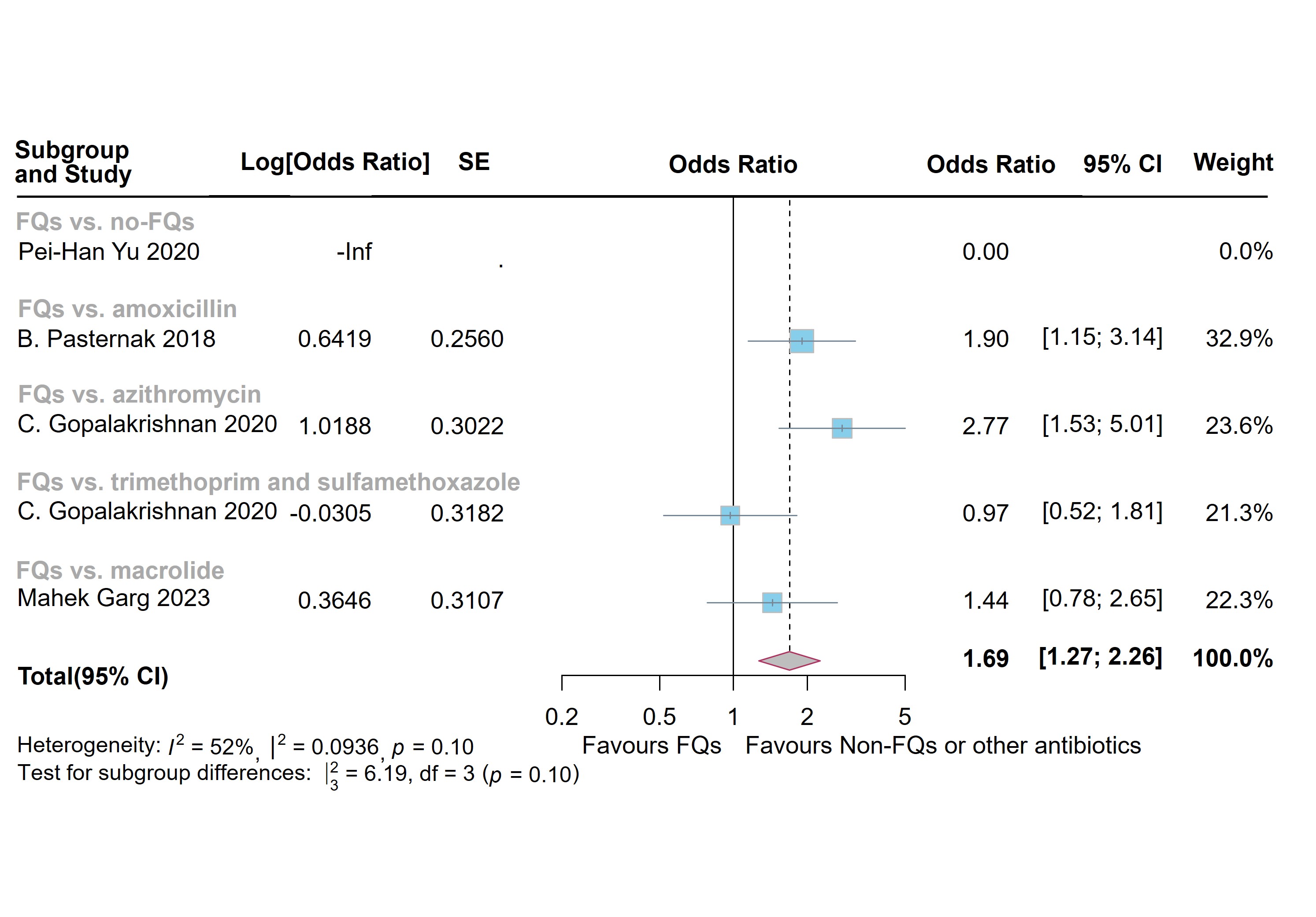

Supplement: Supplementary file 1 [file 2153-8174-27-3-43656-s1.zip › Supplementary Fig. 4.jpg]
